# Supplementary material for: Effect of 24-Week, Late-Evening Ingestion of a Calcium-Fortified, Milk-Based Protein Matrix on Biomarkers of Bone Metabolism and Site-Specific Bone Mineral Density in Postmenopausal Women with Osteopenia
Source: Nutrients. 2022 Aug 24;14(17):3486. doi: 10.3390/nu14173486 (PMC9460355; doi:10.3390/nu14173486)
Supplement: Supplementary file 1 [file nutrients-14-03486-s001.zip › nutrients-1879686-supplementary.pdf]

Supplementary Table S1. Mass and nutrient composition of CON matched for nutrient energy provided by MBPM

Vanilla Flavoured Maltodextrin (CONTROL)

Subject ID	Body mass (kg)	Supplement mass (g)	Energy (kcal·kg-1)	Protein (g·kg-1)	Carbohydrate (g·kg-1)	Fat (g)	Calcium (mg)	Vitamin D (ug)
CON1	76.0	52.1	3	N/A	0.65	N/A	N/A	N/A
CON2	58.0	33.2	2	N/A	0.54	N/A	N/A	N/A
CON3	86.3	52.1	2	N/A	0.57	N/A	N/A	N/A
CON4	88.0	52.1	2	N/A	0.56	N/A	N/A	N/A
CON5	72.7	42.6	2	N/A	0.56	N/A	N/A	N/A
CON6	78.0	52.1	3	N/A	0.63	N/A	N/A	N/A
CON7	68.3	42.6	2	N/A	0.59	N/A	N/A	N/A
CON8	54.9	33.2	2	N/A	0.57	N/A	N/A	N/A
CON9	73.0	42.6	2	N/A	0.55	N/A	N/A	N/A
CON10	84.4	52.1	2	N/A	0.59	N/A	N/A	N/A
CON11	74.8	42.6	2	N/A	0.54	N/A	N/A	N/A
CON12	68.6	42.6	2	N/A	0.59	N/A	N/A	N/A
CON13	63.5	42.6	3	N/A	0.64	N/A	N/A	N/A
CON14	58.4	33.2	2	N/A	0.54	N/A	N/A	N/A
CON15	66.8	42.6	2	N/A	0.61	N/A	N/A	N/A
CON16	53.8	33.2	2	N/A	0.59	N/A	N/A	N/A
CON17	70.2	42.6	2	N/A	0.58	N/A	N/A	N/A
CON18	71.2	42.6	2	N/A	0.57	N/A	N/A	N/A
CON19	113.1	61.6	2	N/A	0.52	N/A	N/A	N/A
CON20	76.6	52.1	3	N/A	0.65	N/A	N/A	N/A
CON21	59.6	33.2	2	N/A	0.53	N/A	N/A	N/A
CON22	53.5	33.2	2	N/A	0.59	N/A	N/A	N/A
CON23	61.5	42.6	3	N/A	0.66	N/A	N/A	N/A
CON24	55.9	33.2	2	N/A	0.56	N/A	N/A	N/A
CON25	72.6	42.6	2	N/A	0.56	N/A	N/A	N/A
CON26	61.2	42.6	3	N/A	0.66	N/A	N/A	N/A
CON27	58.6	33.2	2	N/A	0.54	N/A	N/A	N/A
CON28	66.5	42.6	2	N/A	0.61	N/A	N/A	N/A
CON29	63.6	42.6	3	N/A	0.64	N/A	N/A	N/A
CON30	63.6	42.6	3	N/A	0.64	N/A	N/A	N/A
CON31	87.6	52.1	2	N/A	0.57	N/A	N/A	N/A
CON32	72.3	42.6	2	N/A	0.56	N/A	N/A	N/A
CON33	101.0	61.6	2	N/A	0.58	N/A	N/A	N/A
CON34	67.8	42.6	2	N/A	0.60	N/A	N/A	N/A
CON35	72.8	42.6	2	N/A	0.56	N/A	N/A	N/A
Median	68.6	42.6	2	N/A	0.59	N/A	N/A	N/A
IQR	14.8	9.5	0	N/A	0.05	N/A	N/A	N/A
Min	53.5	33.2	2	N/A	0.52	N/A	N/A	N/A
Max	113.1	61.6	3	N/A	0.66	N/A	N/A	N/A

Supplementary Table S2. Mass and nutrient composition of MBPM providing 0.3g protein per kilogram of body mass.

High Protein Bone Health Powder Instant and Fortified (MBPM)

Subject ID	Body mass (kg)	Supplement mass (g)*	Energy (kcal·kg-1)*	Protein (g·kg-1)	Carbohydrate (g·kg-1)	Fat (g·kg-1)	Calcium (mg)*	Vitamin D (ug)*
MBPM1	69.3	47.8	2	0.32	0.28	0	880	1.0
MBPM2	71.1	47.8	2	0.31	0.27	0	880	1.0
MBPM3	55.7	37.2	2	0.31	0.27	0	684	0.7
MBPM4	80.4	58.4	3	0.34	0.30	0	1075	1.2
MBPM5	59.7	37.2	2	0.29	0.25	0	684	0.7
MBPM6	68.3	47.8	2	0.33	0.28	0	880	1.0
MBPM7	54.1	37.2	2	0.32	0.28	0	684	0.7
MBPM8	68.5	47.8	2	0.33	0.28	0	880	1.0
MBPM9	82.5	58.4	2	0.33	0.29	0	1075	1.2
MBPM10	93	69.0	3	0.35	0.30	0	1270	1.4
MBPM11	54.2	37.2	2	0.32	0.28	0	684	0.7
MBPM12	60.7	47.8	3	0.37	0.32	0	880	1.0
MBPM13	96.3	69.0	3	0.33	0.29	0	1270	1.4
MBPM14	74.6	47.8	2	0.30	0.26	0	880	1.0
MBPM15	48.2	37.2	3	0.36	0.31	0	684	0.7
MBPM16	83.7	58.4	2	0.33	0.28	0	1075	1.2
MBPM17	72.5	47.8	2	0.31	0.27	0	880	1.0
MBPM18	63.5	47.8	3	0.35	0.31	0	880	1.0
MBPM19	77	58.4	3	0.35	0.31	0	1075	1.2
MBPM20	70	47.8	2	0.32	0.28	0	880	1.0
MBPM21	68.9	47.8	2	0.32	0.28	0	880	1.0
MBPM22	85	58.4	2	0.32	0.28	0	1075	1.2
MBPM23	62.2	47.8	3	0.36	0.31	0	880	1.0
MBPM24	58.3	37.2	2	0.30	0.26	0	684	0.7
MBPM25	62	47.8	3	0.36	0.31	0	880	1.0
MBPM26	63.8	47.8	3	0.35	0.30	0	880	1.0
MBPM27	66.3	47.8	3	0.34	0.29	0	880	1.0
MBPM28	43.8	37.2	3	0.40	0.34	0	684	0.7
MBPM29	62	47.8	3	0.36	0.31	0	880	1.0
MBPM30	66	47.8	3	0.34	0.29	0	880	1.0
MBPM31	57.7	37.2	2	0.30	0.26	0	684	0.7
MBPM32	68.2	47.8	2	0.33	0.28	0	880	1.0
**Mean/Median**	67.7	47.8	2	0.33	0.29	0	880	1
**SD/IQR**	12.0	8.0	0	0.02	0.02	0	147	0.2
**Min**	43.8	37.2	2	0.29	0.25	0	684	0.7
**Max**	96.3	69.0	3	0.40	0.34	0	1270	1.4

Note. Median ± IQR was reported for non-normally distributed variables ($p < 0.05$).*-Variables non-normally distributed.
